# Mycophenolate mofetil after tacrolimus for refractory clinically amyopathic dermatomyositis: a case report

**DOI:** 10.3389/fphar.2024.1472667

**Published:** 2024-10-30

**Authors:** Gui-Chen Ling, Chang Su, Ying-Ao Guo, Xia Qiu, Jia-Wei Liu, Min Xiao, Yu-Ya Xiao, Shuo Yang, Jian-Yong Zhang, Jing-Jing Xie

**Affiliations:** ^1^ The Fourth Clinical Medical College of Guangzhou University of Chinese Medicine, Shenzhen, Guangdong, China; ^2^ The Department of Rheumatology, Shenzhen Traditional Chinese Medicine Hospital, Shenzhen, Guangdong, China; ^3^ The Department of Traditional Chinese Medicine, The University of Hong Kong-Shenzhen Hospital, Shenzhen, Guangdong, China

**Keywords:** anti-MDA5-associated dermatomyositis, case report, rapidly progressive interstitial lung disease, mycophenolate mofetil, clinically amyopathic dermatomyositis (CADM)

## Abstract

Dermatomyositis (DM) positive for anti-melanoma differentiation-associated gene 5 (MDA5) antibodies, mainly when linked with rapidly progressive interstitial lung disease (RP-ILD), is considered a refractory disease. Our report describes a critical case of clinically amyopathic dermatomyositis (CADM) with RP-ILD that tested positive for both anti-MDA5 and anti-Ro-52 antibodies. The patient showed a limited response to a combined therapy regimen of prednisone, iguratimod, and tacrolimus. However, after adjunct therapy with mycophenolate mofetil (MMF), the patient’s condition was controlled, his serum KL-6 levels decreased, and anti-MDA5 antibodies became negative. During the 68-week follow-up, the patient’s condition remained stable, with a satisfactory quality of life. This report also discusses the potential role of inflammatory cytokines in the pathophysiology of CADM and RP-ILD. Further research is required to confirm these results and investigate the application of MMF in maintenance therapy for CADM-associated RP-ILD.

## Introduction

Clinically amyopathic dermatomyositis (CADM), characterized by anti-melanoma differentiation-associated protein 5 (anti-MDA5) antibodies, represents a distinct subgroup within the spectrum of idiopathic inflammatory myopathies (Hallowell and Danoff, 2023). This rare autoimmune disorder primarily manifests with pronounced dermatological symptoms, while muscle involvement remains minimal or absent. A notable feature of this clinical phenotype is the frequent development of rapidly progressive interstitial lung disease (RP-ILD), which significantly influences patient prognosis (Lu et al., 2024). Additionally, anti-Ro-52 antibodies are frequently detected alongside antibodies associated with myositis. Anti-Ro52 antibodies are commonly found in patients with MDA5 antibody-positive dermatomyositis (MDA5-DM), and elevated levels of these antibodies are associated with decreased survival rates (Wang et al., 2023).

The etiology and pathogenesis of anti-MDA5-associated RP-ILD are not well understood, and management typically requires a regimen of corticosteroids combined with immunosuppressive agents. However, there is a lack of evidence-based medicine to guide the selection of specific immunosuppressants. Calcineurin inhibitor, often combined with glucocorticoids, are perceived as the standard initial therapy (Romero-Bueno et al., 2020). When these inhibitors are contraindicated or ineffective, individualized decisions to switch or supplement treatments may be necessary. For refractory patients, various immunosuppressive regimens have been trialed, including intravenous immunoglobulin therapy and rituximab, with varying success rates (Shirai et al., 2023; Wang et al., 2022). Additionally, plasmapheresis and polymyxin B hemoperfusion are viable salvage therapies (Saito et al., 2021). Notably, MMF serves as a salvage therapy in some rare diseases, including dermatomyositis and IgA nephropathy (Bhat et al., 2023). We present a case in which a treatment plan combining MMF was successful and provided a viable option for patients with anti-MDA5-associated RP-ILD when a change in treatment was necessary.

## Case report

**FIGURE 1:**
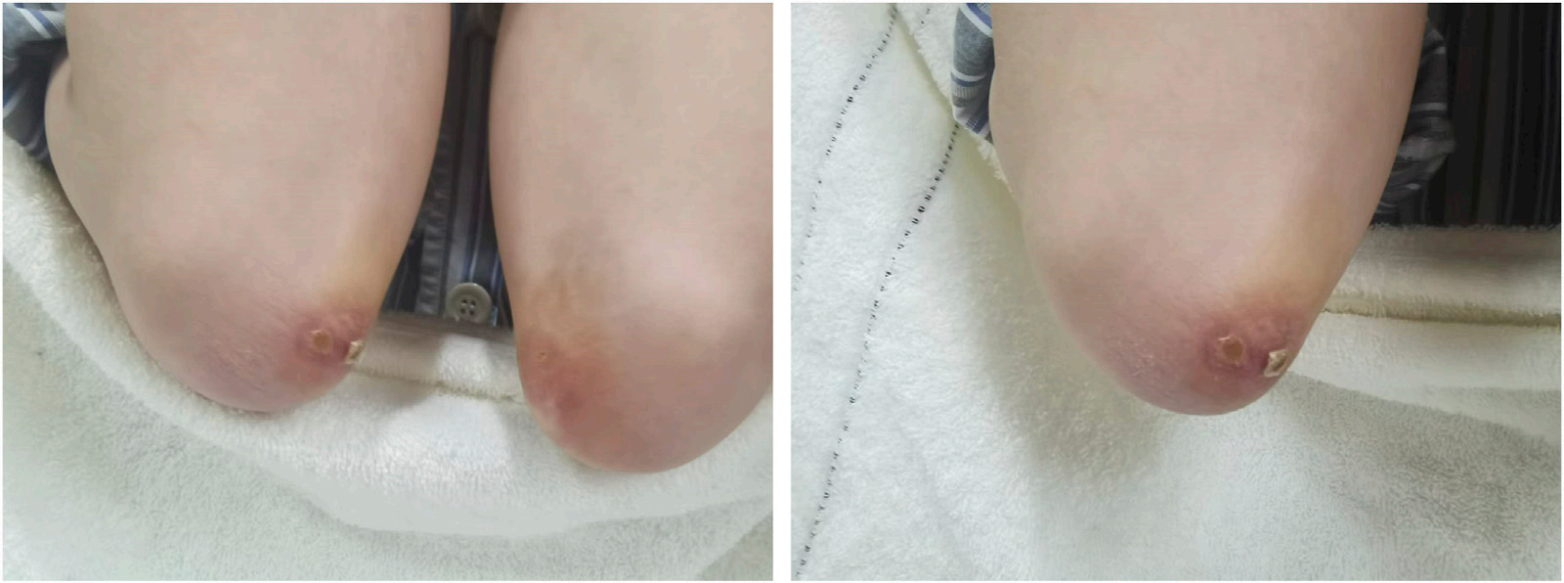
Gottron’s papules on the extensor surfaces of the elbows. Erythematous, scaly, and slightly raised papules are present bilaterally over the elbows, consistent with Gottron’s papules, a characteristic cutaneous manifestation of dermatomyositis.

**FIGURE 2:**
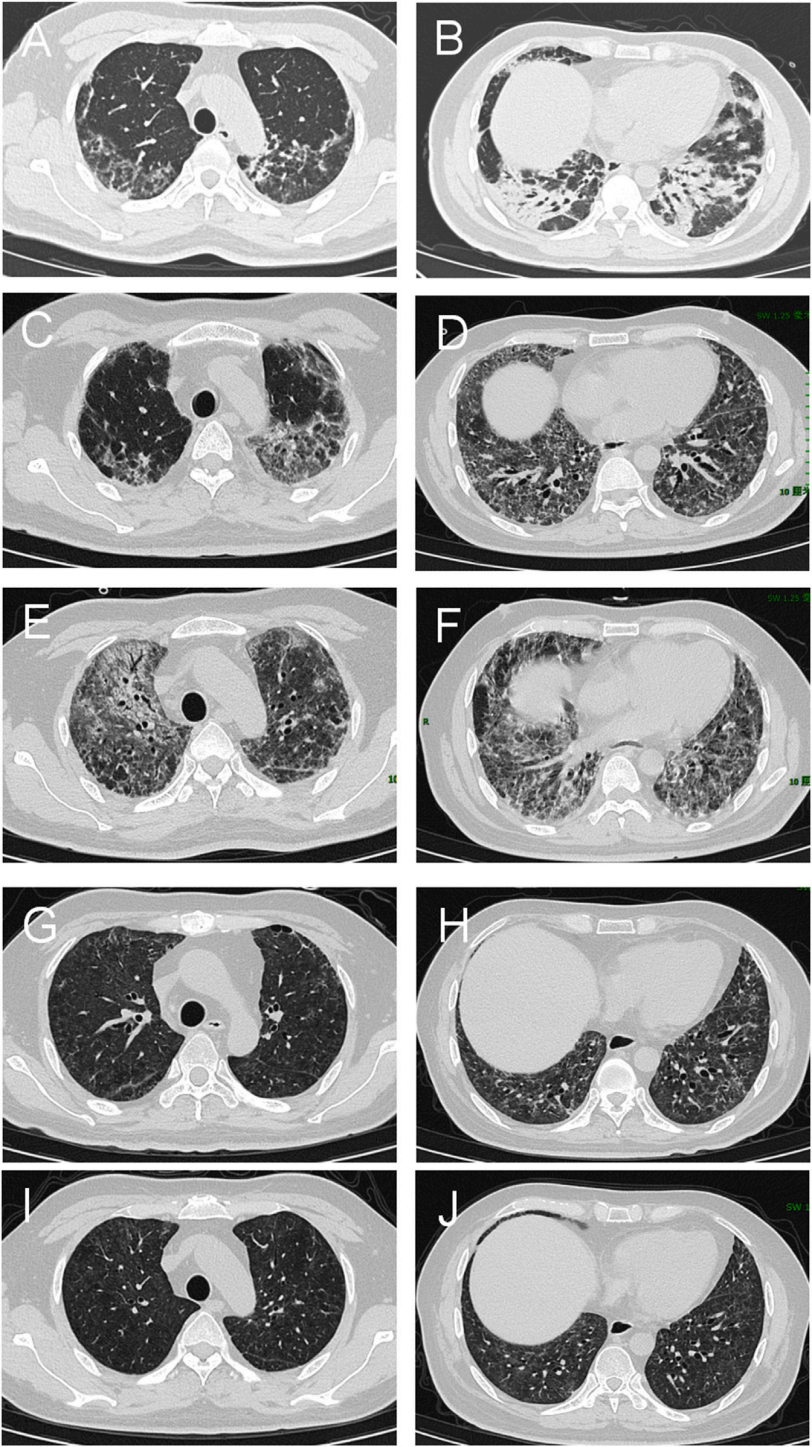
Chest HRCT of the patient with interstitial lung disease associated with MDA5-DM. **(A, B)** Baseline CT scans reveal extensive bilateral ground-glass opacities and consolidation in the lungs, indicating active disease. **(C, D)** Follow-up scans at 4 weeks indicate a slight reduction in ground-glass opacities but persistent consolidation, indicating partial improvement. **(E, F)** By 12 weeks, HRCT shows significant disease progression characterized by widespread bilateral consolidations, persistent ground-glass opacities, and newly developed reticular opacities, suggestive of advancing interstitial fibrosis. **(G, H)** At 44 weeks, significant resolution of ground-glass opacities and consolidation, with further fibrotic changes indicating chronic scarring. **(I, J)** By 68 weeks, there is continued improvement with minimal residual ground-glass opacities and consolidation. Fibrotic changes remain stable, indicating a near-complete response to treatment and stabilization of chronic changes.

A 36-year-old Chinese male presented with symptoms including fever, lateral finger papules, cough, and shortness of breath lasting for 1 month. He had been previously healthy, with no significant past medical history, family history of autoimmune diseases, smoking history, or occupational exposure to dust or chemicals. He worked as an office clerk and denied any illicit drug use. The patient was initially diagnosed with suspected pneumonia at a community hospital and did not respond to empiric treatment with imipenem or levofloxacin. Subsequently, he developed chest pain and worsening dyspnea, necessitating referral and immediate admission to our hospital. The patient was afebrile with a 120/82 mmHg blood pressure on examination. His respiratory and pulse rates were significantly elevated at 36 breaths per minute and 118 beats per minute, respectively, with a critically low oxygen saturation of 50%. Dermatological examination revealed erythematous to violaceous papules over the extensor surfaces of the elbow joints, consistent with Gottron’s papules(Figure 1). Additionally, he had hyperkeratotic, rough skin on the lateral aspects of the fingers and palms, resembling “mechanic’s hands.” There was no muscle tenderness or weakness on manual muscle testing. Sputum bacterial and fungal cultures were negative. Additionally, tests for influenza virus and SARS-CoV-2 were also negative, further ruling out common viral etiologies. Laboratory tests revealed a normal white blood cell count of 6.7 × 10^9/L. Creatine kinase (CK) was within normal limits at 85 U/L, and lactate dehydrogenase (LDH) was 230 U/L. Alanine aminotransferase (ALT) and aspartate aminotransferase (AST) levels were 20.4 U/L and 17.5 U/L, respectively, both within normal range. The arterial blood gas analysis revealed a pH of 7.457, a partial pressure of oxygen (PaO_2_) of 54 mmHg, and a partial pressure of carbon dioxide (PaCO_2_) of 41.7 mmHg, indicating hypoxemia with respiratory alkalosis. The erythrocyte sedimentation rate (ESR) was 13 mm/h, and C-reactive protein (CRP) was 3.14 mg/L, both within normal limits. Notably, serum ferritin was elevated at 666.3 μg/L, and Krebs von den Lungen-6 (KL-6) was significantly increased to 2,665 U/mL, suggesting alveolar epithelial cell injury. A myositis-specific antibody panel revealed strong positivity for anti-MDA5 and anti-Ro-52 antibodies, with other autoantibodies, including antinuclear antibodies (ANA), being negative. Due to the patient’s severe hypoxemia and respiratory distress, pulmonary function tests were not feasible at admission. High-resolution computed tomography (HRCT) of the chest demonstrated diffuse bilateral ground-glass opacities and reticular patterns, consistent with interstitial lung disease (ILD) (Figures 2A, B).

Based on the characteristic cutaneous findings without obvious muscle symptoms, the presence of anti-MDA5 antibodies, and HRCT evidence of ILD, the patient was diagnosed with anti-MDA5 antibody-positive CADM complicated by RP-ILD[(Kurtzman and Vleugels, 2018; Gerami et al., 2006; Stonecipher et al., 1993)]. Prior to this diagnosis, we excluded other potential causes including pneumonia, viral infections, lung cancer progression, tuberculosis, and heart failure. Given the severity of his condition, the patient was started on high-flow oxygen therapy at 40 L/min with an FiO_2_ of 80% to correct hypoxemia. The combination therapy included intravenous methylprednisolone at 160 mg once daily (gradually tapered to 40 mg once daily) and tacrolimus at 1 mg twice daily as induction therapy. Intravenous immunoglobulin was administered as an adjunctive treatment at a dose of 20 g daily for 5 days. Additionally, the patient received oral nintedanib at 100 mg twice daily for anti-fibrotic treatment. Trimethoprim–sulfadiazine was also administered as prophylaxis for opportunistic infections. After 4 weeks of treatment, the patient was discharged under continuous nasal oxygen supplementation (3 L/min). All symptoms had resolved except for exertional oxygen desaturation. Follow-up chest HRCT showed a slight reduction in ground-glass opacities but persistent consolidation, indicating partial improvement (Figures 2C, D). The treatment regimen was adjusted to methylprednisolone 30 mg once daily, iguratimod 25 mg twice daily, tacrolimus 1 mg twice daily, and nintedanib 100 mg twice daily.

After 12 weeks of treatment, the patient’s respiratory condition deteriorated, necessitating readmission. He presented with worsening chest tightness and shortness of breath. Laboratory tests revealed a markedly elevated serum KL-6 level of 9500 U/mL and ferritin of 739 μg/L. Follow-up chest HRCT demonstrated significant disease progression, characterized by widespread bilateral consolidations, persistent ground-glass opacities, and newly developed reticular patterns. While no signs of infection were present, these findings indicated advancing interstitial fibrosis (Figures 2E, F). The treatment regimen was adjusted to prednisone pulse and tacrolimus, followed by conversion from tacrolimus to MMF 1500 mg once daily, continuing oral prednisolone 50 mg once daily, iguratimod 25 mg twice daily, and nintedanib 100 mg twice daily. After treatment, the patient’s condition improved, with mild dyspnea during daily activities and difficulty breathing only during exertion. He was discharged on supplemental oxygen at 3 L/min, with an SpO_2_ of 96%. The patient continued home oxygen therapy for 2 months following this adjustment. Chest tightness and shortness of breath gradually improved, and by 20 weeks of treatment, the patient no longer required supplemental oxygen, with an SpO_2_ of 99%. Follow-up testing revealed that the MDA-5 antibody titer had turned negative.

During the 44 weeks, the patient was in remission. HRCT showed interstitial changes in both lungs and bronchiectasis with scattered exudates (Figures 2G, H). Consequently, the treatment regimen was adjusted to oral prednisone 15 mg once daily, MMF 1000 mg once daily, iguratimod 25 mg twice daily, and nintedanib 100 mg twice daily. At the 68-week follow-up, the patient’s medication was further modified. He continued taking MMF, now reduced to 750 mg once daily, while prednisone was gradually tapered to 5 mg once daily. Iguratimod was decreased to 25 mg once daily, and nintedanib was discontinued. The patient’s condition was well controlled, with no recurrence of the disease. Laboratory tests revealed that the serum ferritin levels, and inflammatory marker levels returned to normal. Follow-up chest HRCT revealed radiological improvement (Figures 2I, J). According to pulmonary function tests, the predicted forced vital capacity (FVC) was 76%, and the lung diffusion capacity was 57%.

**FIGURE 3:**
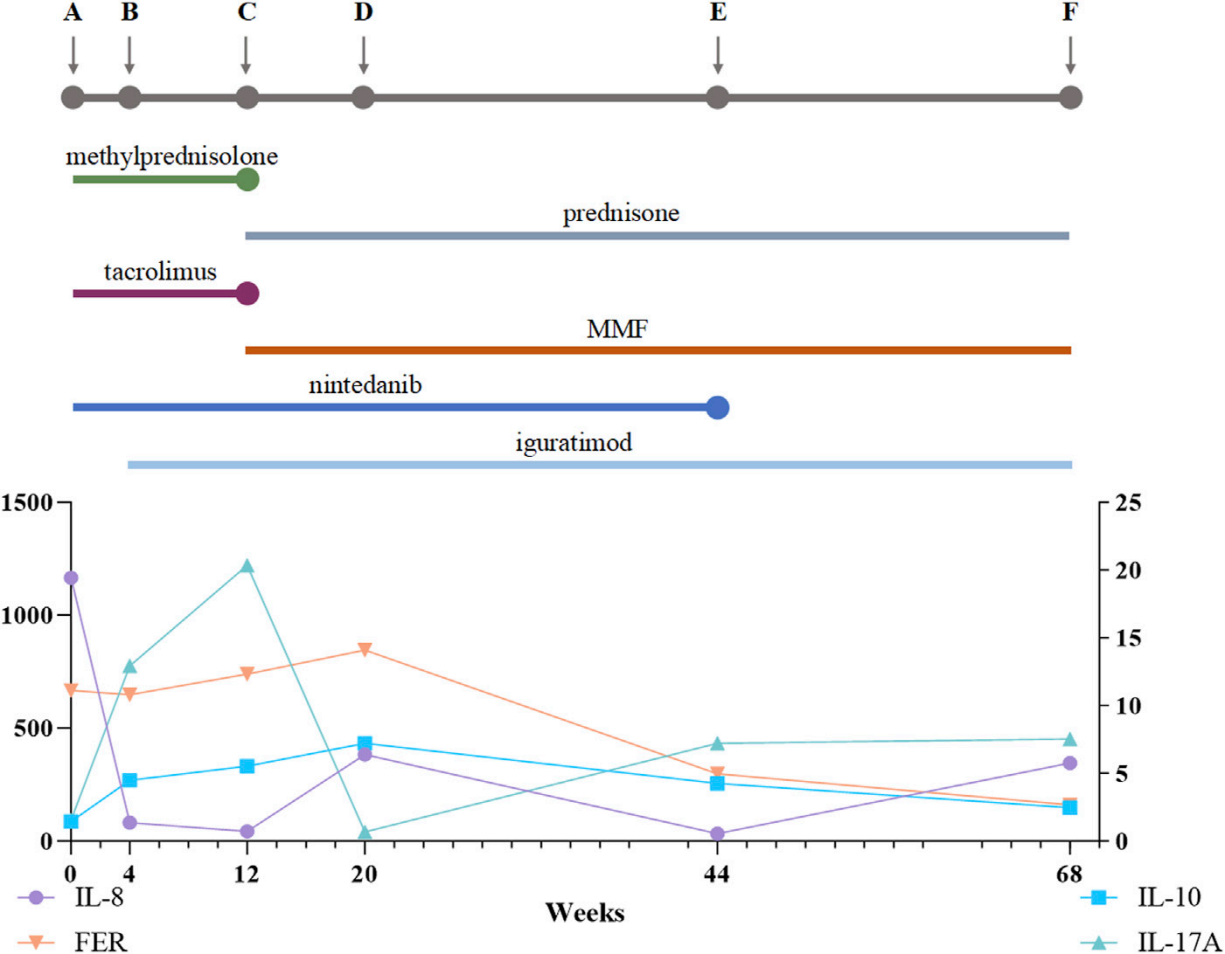
Treatment Process and Cytokine Changes. **(A)** Prior to treatment; **(B)** 4 weeks after treatment with a methylprednisolone pulse followed by oral methylprednisolone (40 mg qd), oral tacrolimus (1 mg bid), and nintedanib at 100 mg bid; **(C)** 12 weeks after treatment with a prednisone pulse and tacrolimus and then conversion from tacrolimus to MMF 1500 mg qd, continuing to oral prednisone 50 mg qd, iguratimod 25 mg bid, and nintedanib 100 mg bid; **(D)** 20 weeks after treatment, oral prednisone 40 mg qd, MMF 1000 mg qd, iguratimod 25 mg bid, and nintedanib 100 mg bid; **(E)** 44 weeks after treatment, oral prednisone 15 mg qd, MMF 1000 mg qd, iguratimod 25 mg bid, and nintedanib 100 mg bid; **(F)** 68 weeks after treatment, oral prednisone 5 mg/d, MMF 750 mg qd, and iguratimod 25 mg qd. MMF, mycophenolate mofetil. The line chart shows IL-8, FER, IL-10, and IL-17A levels over the 68-week treatment period.

We measured serum levels of several cytokines (IL-8, IL-10, IL-17A) and ferritin before and throughout this patient’s treatment regimen (Figure 3). We also evaluated serum concentrations of other cytokines, including IL-1β, IL-2, IL-4, IL-5, IL-6, IL-12(p70), IFN-α, IFN-γ, and TNF-α (Table 1).

**TABLE 1:** Changes of serum cytokine levels before and during treatment.

Time of conversion (weeks)	Serum cytokine level (pg/mL)												FER (μg/L) 23.9–336.2	KL-6 (U/mL) 0–500
	IL-1β 0–12.4	IL-2 0–5.71	IL-4 0–3.0	IL-5 0–3.1	IL-6 0–5.3	IL-8 0–20.6	IL-10 0–4.91	IL12 (P70) 0–3.4	IL-17A 0–20.6	TNF-α 0–4.6	IFN-α 0–8.5	IFN-γ0-7.42		
0	3.45	0.75	0.79	0.49	5.53	1,165.08	1.45	3.08	1.55	2.11	2.79	0.32	666.3	2,665
4	1.06	1.28	0.60	1.16	8.91	81.45	4.48	1.30	12.93	0.88	0.70	1.65	648.4	3,894
12	2.47	2.81	1.66	3.09	4.33	43.21	5.54	3.35	20.34	3.96	3.98	2.35	739	9,500
20	1.43	1.25	0.92	1.06	4.60	383.57	7.22	1.95	0.68	1.21	2.96	2.69	845	>10,000
44	2.17	0.32	1.55	0.75	5.00	32.74	4.25	1.72	7.21	1.17	0.74	2.52	297.5	ND
68	0.94	0.17	2.04	1.27	3.05	345.66	2.47	2.30	7.54	3.13	0.96	2.84	160.5	1,504

Abbreviations: m, month; IL-1β, Interleukin-1, beta; IL-2, Interleukin-2; IL-4, Interleukin-4; IL-5, Interleukin-5; IL-6, Interleukin-6; IL-8, Interleukin-8; IL-10, Interleukin-10; IL12, Interleukin-12; IL-17A, Interleukin-17A; TNF-α, tumor necrosis factor alpha; IFN-α, interferon alpha; IFN-γ, interferon gamma.

## Discussion

MDA5-DM presents as a rapidly advancing and aggressive disease. It is highly likely that patients will develop RP-ILD, which is associated with high mortality (Lu et al., 2024). While high-dose corticosteroids combined with immunosuppressants may benefit these patients (McPherson et al., 2022), the use of immunosuppressants remains largely empirical. We report the efficacy of a combined therapeutic approach, which included transitioning from tacrolimus to MMF, in treating a patient with life-threatening RP-ILD associated with anti-MDA5 antibody-positive CADM. Our patient was admitted with positive tests for anti-MDA5 and anti-Ro-52 antibodies. Elevated ferritin and KL-6 levels, along with respiratory impairment, were also observed, raising concerns about the patient’s prognosis.

Despite rapidly developing refractory respiratory failure, the patient initially responded well to combination therapy consisting of methylprednisolone, tacrolimus, and supplementary intravenous immunoglobulin, along with maximum respiratory support and additional treatments including trimethoprim–sulfadiazine and nintedanib. Antifibrotic therapy may be beneficial for MDA5-DM-associated ILD (Flaherty et al., 2019). Increasing evidence suggests that the antifibrotic drug nintedanib helps slow the progression of ILD and reduce the frequency of acute exacerbations (Lamb, 2021). After being discharged from the hospital in stable condition, he later experienced recurrences with worsening lung involvement. CT scans indicated progressive interstitial lung disease, and the patient still faced a risk of fulminant respiratory failure. The patient and family refused plasma exchange as adjunctive therapy. However, due to the toxicity associated with cyclophosphamide therapy, they did not want to use it as a first-line treatment.

MMF is an immunosuppressive agent that acts by inhibiting inosine monophosphate dehydrogenase, the rate-limiting enzyme in the *de novo* synthesis of guanine nucleotides (Allison and Eugui, 2000). This inhibition leads to a reduction in guanosine monophosphate levels, ultimately suppressing the proliferation of T and B lymphocytes, which rely heavily on the *de novo* pathway for purine synthesis due to their lack of a salvage pathway. By reducing lymphocyte proliferation, MMF decreases the production of autoantibodies and inflammatory cytokines, which are central to the pathogenesis of MDA5-DM. In MDA5-DM, the presence of anti-MDA5 autoantibodies suggests a pivotal role of B cells in the disease’s pathophysiology (Coutant et al., 2022). Additionally, T lymphocytes contribute to immune-mediated tissue damage, particularly in the lungs, leading to RP-ILD (Takahisa et al., 2010). By targeting both T and B lymphocyte proliferation, MMF addresses the underlying autoimmune processes driving disease progression. It has demonstrated effectiveness in the treatment of MDA5-DM patients with ILD (Hata et al., 2024). MMF has demonstrated efficacy in resolving respiratory failure in patients (Paul et al., 2016). It offers advantages over other drugs used for refractory conditions (such as rituximab), being more cost-effective and easier to administer. Considering the patient’s condition and drawing on our extensive experience, we chose MMF as a secondary steroid-sparing agent after tacrolimus treatment. With informed consent, we replaced tacrolimus with MMF. After 20 weeks, the patient’s MDA-5 antibody titer became negative, and his symptoms significantly improved. However, since MMF was used after tacrolimus treatment, it is difficult to determine which drug was responsible for the conversion of anti-MDA5 antibodies. Nevertheless, the combination of MMF plays a significant role in maintenance therapy. Therefore, we believe that MMF has potential in the maintenance treatment of RP-ILD patients with MDA5-DM.

The mechanism by which MDA5-DM affects RP-ILD, including autoimmunity, has not yet been fully elucidated. Excessive activation of monocytes and macrophages may play a significant role in the pathophysiology of this condition (Shirakashi et al., 2020). High cytokine levels are present in 90% of individuals who succumb to PM/DM-ILD (Matsuda et al., 2020). Research indicates that ILD disease activity in PM/DM patients is associated with increased levels of the cytokines TNF-α, IL-6, and IL-8 (Gono et al., 2014). Additionally, the levels of interferon -related cytokines, such as IFN-α, IFN-γ, and IP-10, are significantly elevated in the anti-MDA5 subset compared to the anti-TIF1γ subset, underscoring the potential role of these cytokines in disease differentiation (Bai et al., 2021).

We found that IL-2, IL-5, IL-1β, IFN-α, and TNF-α decreased after 1 month of Tac treatment. Although iguratimod is not the central focus of this case, we included it in the treatment regimen based on studies indicating its potential protective role in interstitial lung disease (Han et al., 2021). Specifically, due to the elevated levels of pro-inflammatory cytokines (such as IFN-γ, IL-6, and IL-17A) observed in our patient, iguratimod was added empirically to help modulate the immune response and alleviate lung inflammation (Xu et al., 2015). After initiating MMF treatment, the levels of inflammatory factors rapidly decreased, coinciding with disease remission. IL-8, IL-10, and IL-17A levels are of particular significance. IL-8 is secreted primarily by monocytes, T lymphocytes, neutrophils, and various other cells upon stimulation and initiates acute inflammatory responses, leading to a cytokine storm (Li J. et al., 2021; Harada et al., 1994). Elevated IL-8 levels are correlated with acute lung injury and increased mortality risk in patients with severe COVID-19 (Li J. et al., 2021; Li H. et al., 2021). In anti-MDA5-related ILD, high IL-8 levels are linked to disease activity and fatal outcomes (Gono et al., 2014). In our case, IL-8 levels were elevated to 1,165.08 pg/mL during the acute phase, indicating acute inflammation. Elevated cytokine levels did not align with disease progression or treatment response. Serum ferritin and KL-6 levels offer significant predictive value for ILD in patients with MDA5-DM (Gono et al., 2010; Ye et al., 2019). Elevated IL-10 levels were correlated with KL-6 and ferritin levels, consistent with previous research (Kawasumi et al., 2014). A continuous increase in the IL-10 concentration until symptom remission and a negative MDA-5 antibody titer suggested that the initial treatment was inadequate for calming inflammation. Additionally, the role of inflammatory cytokines in the pathophysiology of MDA5-DM and their potential as biomarkers for disease activity and treatment response merit further investigation.

## Conclusion

In conclusion, we reported a life-threatening case of dermatomyositis with both Ro-52 and MDA5 antibodies complicated by RP-ILD. Despite initial aggressive immunosuppressive therapy, the patient’s pulmonary condition continued to deteriorate. The switch to MMF ultimately improved respiratory failure and facilitated the conversion of anti-MDA5 antibodies. Although further studies are necessary to confirm our findings, MMF may represent a valuable option for maintenance therapy in patients with this challenging condition.

## Data Availability

The original contributions presented in the study are included in the article/supplementary material, further inquiries can be directed to the corresponding authors.
